# Robust IoT-based nursing-care support system with smart bio-objects

**DOI:** 10.1186/s12938-018-0582-5

**Published:** 2018-11-06

**Authors:** Cheng-Fa Chiang, Fang-Ming Hsu, Kuo-Hui Yeh

**Affiliations:** 1 Department of Information Management, National Dong Hwa University, Hualien, 97401 Taiwan, ROC; 2 Physical Education Center, National Dong Hwa University, Hualien, 97401 Taiwan, ROC

## Abstract

**Background:**

The significant advancement in the mobile sensing technologies has brought great interests on application development for the Internet-of-Things (IoT). With the advantages of contactlessness data retrieval and efficient data processing of intelligent IoT-based objects, versatile innovative types of on-demand medical relevant services have promptly been developed and deployed. Critical characteristics involved within the data processing and operation must thoroughly be considered. To achieve the efficiency of data retrieval and the robustness of communications among IoT-based objects, sturdy security primitives are required to preserve data confidentiality and entity authentication.

**Methods:**

A robust nursing-care support system is developed for efficient and secure communication among mobile bio-sensors, active intelligent objects, the IoT gateway and the backend nursing-care server in which further data analysis can be performed to provide high-quality and on-demand nursing-care service.

**Results:**

We realize the system implementation with an IoT-based testbed, i.e. the Raspberry PI II platform, to present the practicability of the proposed IoT-oriented nursing-care support system in which a user-friendly computation cost, i.e. 6.33 ms, is required for a normal session of our proposed system. Based on the protocol analysis we conducted, the security robustness of the proposed nursing-care support system is guaranteed.

**Conclusions:**

According to the protocol analysis and performance evaluation, the practicability of the proposed method is demonstrated. In brief, we can claim that our proposed system is very suitable for IoT-based environments and will be a highly competitive candidate for the next generation of nursing-care service systems.

## Background

With the rapid growth of information and communications technologies, such as Bluetooth Low Energy (BLE), 3G/4G/5G and NFC/RFID, a comprehensive evolution of the Internet has given rise to a ubiquitous network consisting of mobile intelligent objects, called the Internet of Things (IoT). In IoT-based environments, “contactless data sensing” and “collecting and information analyzing and retrieving” are fundamental components for the provision of human value-added services in a more transparent and faster way than before. Among these services, in particular, the development of IoT-oriented nursing-care service systems are one the most promising and important directions, and are therefore a major focus of government and industry. A nursing-care service system is exploited for data collection, data storing, data retrieval and information display needed in nursing activities via modern information and communication technologies. With the advantages of contactlessness and efficiency brought by the data retrieval on intelligent IoT-objects, innovative types of on-demand nursing-care service systems have promptly been developed in these years. Meanwhile, the issue of system security and patient privacy has been focused by governments and research community. The potential to reveal patient privacy and system security vulnerability may exist wherever personally identifiable information is collected, processed, or stored in a hospital information system. Based on our survey, we present the major principles during patient private data processing: (1) be processed and used for lawful purposes; (2) unauthorized or unlawful processing must be measured; (3) accountability is required; (4) consent for data processing must be guaranteed; (5) be processed with an adequate level of protection and (6) adequate and relevant to the purpose for which it is processed.

In this paper, we present a robust IoT-based nursing-care support system in which fixed environmental sensing objects and intelligent smart objects are deployed in the field and on patients, respectively, to support high-quality nursing-care service. To satisfy the security and privacy requirements, we argue that sturdy cryptographic primitives must be implemented on IoT-objects to construct robust communications among entities. Nevertheless, based on current semiconductor technology, most of IoT-objects cannot afford heavy cryptographic primitives, such as asymmetric cryptography, due to limited computational resources. Therefore, a refinement of the traditional secure communication scheme should be launched in terms of the performance standpoint. That is, we have to thoroughly consider the trade-off between efficiency and the robustness of the adopted cryptographic components to appropriately meet the hardware limitation of IoT-objects and the security requirements we need. According to the analyses, the robust cryptographic module with a reasonable and acceptable computation cost, i.e. SHA-384, SHA-215 and SHA-3 [1, 2], will be good candidate techniques to simultaneously satisfy the security and performance requirements. In conclusion, we would like to demonstrate an efficient IoT-based communication mechanism for nursing-care service systems in which SHA-3 are mainly adopted as the major data protection technique to simultaneously achieve system security and patient privacy during the operation of the proposed system.

In the following, we present the state of the art of IoT application and security. In 2012, Jara et al. [3] proposed an IoT-based knowledge acquisition and management platform. This platform is composed of two parts, i.e. a wireless transmission of continuous vital signs through 6LoWPAN and a patient identification through RFID. The presented system also adopted a data analysis model and pre-processing module for patient health management. Next, Berhanu et al. [4] introduced an e-Health system with IoT devices in which a robust security scheme is included. The authors investigated the impact of antenna orientation on energy consumption to examine the validation of the proposed system. The issue of scalability is also studied through the feasibility of embedding the lightweight security solutions into the ASSET (adaptive security for smart Internet of Things in e-health) [5]. Later, Torjusen et al. [6] verified that an enabler integrating into the ASSET adaptive security framework and provided an e-healthcare security framework via the IoT. Critical requirements for run-time verification are presented as formal specifications. After that, Bello and Zeadally [7] proposed an intelligent routing cooperation scheme for device-to-device communication. The operation of different network standards in the case of intermittent connection is considered in which the device will be affected by it’s limited resources. In 2016, Gope and Hwang [8] proposed an IoT application system for healthcare on body sensor networks (BSN), called BSN-Care, which is able to provide effective real-time monitoring, patient information management and security needs for healthcare. In order to achieve the claimed services, a comprehensive integration of clinical devices and efficient collection of data are demonstrated. Note that the authors also introduced a similar concept for secure communication on IoT in [9].

Yao et al. [10] modified the fast one-way accumulator, proposed by Nyberg [11], to build a lightweight multicast authentication mechanism. However, the proposed method is only applicable to the small-scale IoT networks. It is limited by it’s scalability. After that, Ning et al. [12] demonstrated an aggregation-based hierarchical authentication scheme. This method provides a strong security and is applicable to the U2IoT architecture. The main idea is to establish backward and forward anonymous data transmission among multiple targets. In addition, various techniques, i.e. directed path descriptors, homomorphism functions, and Chebyshev chaotic maps, are jointly applied for mutual authentication. Later, Hernández-Ramos et al. [13] proposed a framework for lightweight authentication and authorization. This presented framework is based on the reference model proposed by the EU FP7 IoT-A project. Meanwhile, Kawamoto et al. [14] proposed a location-based authentication system, where ambient information from IoT-based sensors are collected and analyzed as the authentication tokens. To pursue high authentication accuracy, the proposed system automatically and continuously adjusts the system parameters according to surrounding environment situation. Next, Cirani et al. [15] proposed an IoT-OAS architecture with the characteristics of flexible, highly configurable, and easy-to-integrate with existing services. The authors further provide an authorization platform which can invoke an external OAuth-based authorization service. The evaluation of the proposed architecture is based on Contiki OS-based constrained devices. In 2017, Cha et al. [16] demonstrated a privacy-aware mechanism for secure communication and efficient access-control among BLE-based devices. The proposed mechanism is based on elliptic curve cryptography. In addition, the authors presented a framework for the management of security examination reports of BLE-based applications.

## Methods

This section introduces the underlying IoT communication architecture and then presents the proposed nursing-care support system consisting of a registration process and an authentication process.

### Targeted IoT communication scenario

**Fig. 1:**
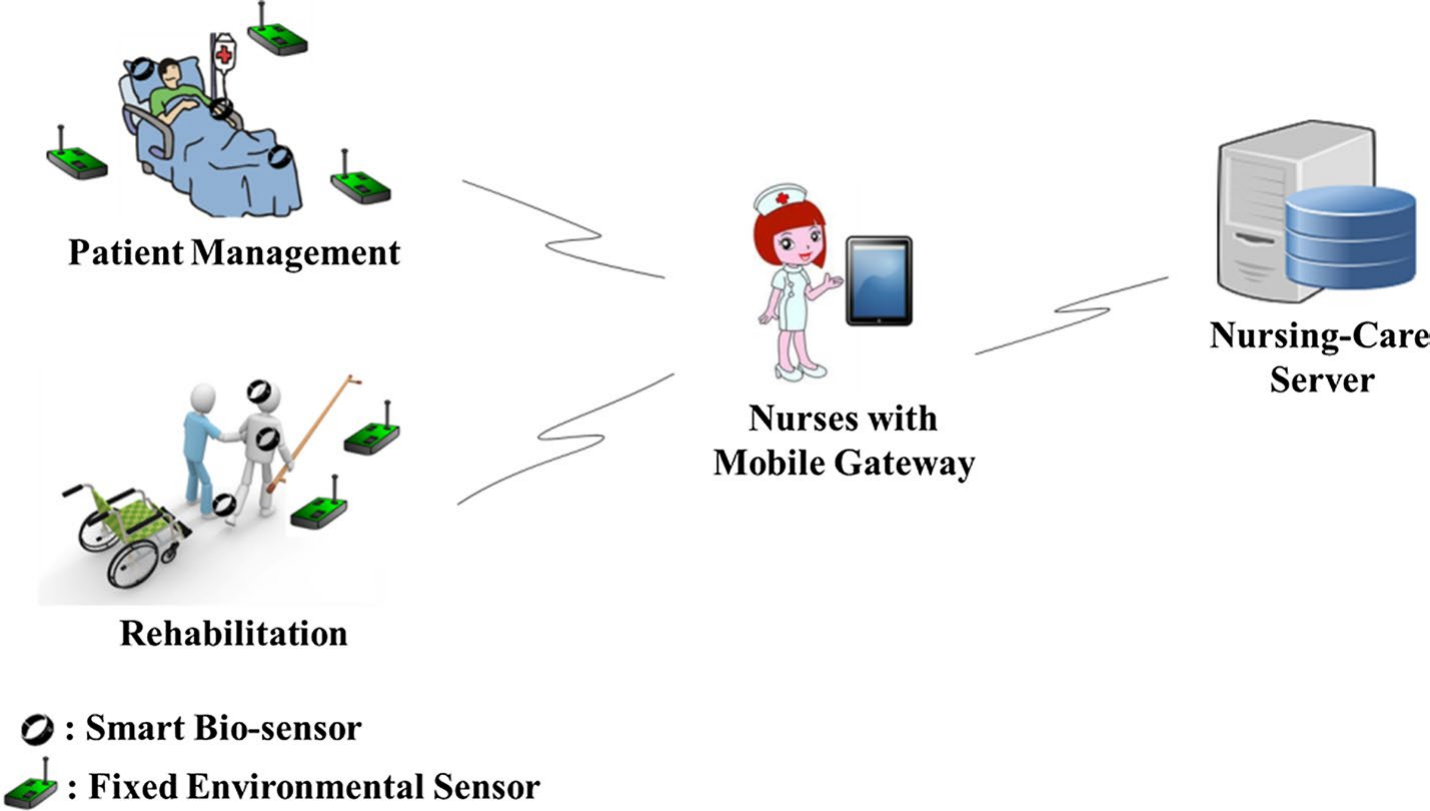
The targeted IoT communication architecture

In this section, we introduce the underlying IoT communication architecture of our proposed nursing-care support system. Figure 1 demonstrates the scenario we target on, i.e. patient management and rehabilitation, in which fixed environmental sensors and medical sensing devices are deployed on field and on the patient, respectively, to support caregivers (such as nurse) in activities of patient care and rehabilitation. Three important components are existed in the identified IoT communication scenario, i.e. a backend nursing-care server, a mobile gateway (usually handheld by the caregiver), and intelligent devices (such as fixed sensor nodes or medical sensing devices). The intelligent devices are utilized for sensing and collecting environmental parameters and patients’ bio-data, while the caregiver will operate a handheld smart device as the mobile gateway to communicate with the intelligent devices. Note that the patient’s bio-data are such as electrocardiography, electroencephalography, electromyography and blood pressure retrieved from the patient. After that, with the environmental data and the patient’s bio-data, the caregiver can identify patient’s need in a faster way. More accurate and timely treatment services will then be able to deliver to the patients.

### The IoT-based nursing-care support system

**Fig. 2:**
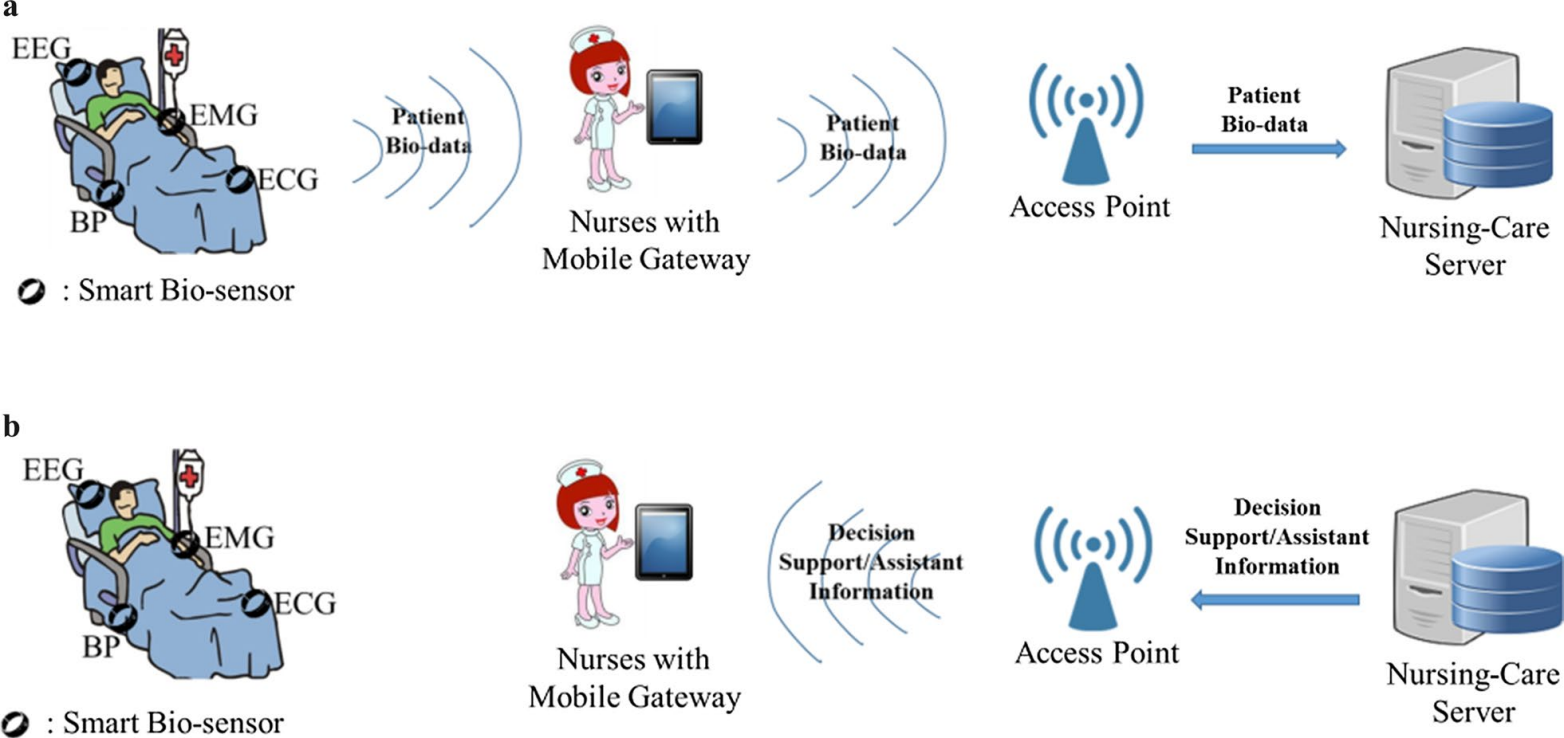
The proposed IoT-based nursing-care support system

In this section, we present the proposed nursing-care support system in which IoT-based intelligent devices are adopted on the patient and the corresponding environment (e.g., Fig. 2). From Fig. 2a, the nurse first utilizes his/her handheld mobile gateway to retrieve the data from smart bio-objects. All of the retrieved data will be forwarded to the backend nursing-care server. At the backend server, a further data analysis for the mining of the patient’s needs will be performed. Then, in Fig. 2b, decision support/assistant information will be derived and delivered to the nurse to support better-quality nursing care services on the patient.

In general, the proposed system consists of two phases, i.e. the registration phase and the authentication phase. In the registration phase, the security credentials will be securely agreed and shared among the communication entities, i.e. intelligent devices (smart bio-objects and fixed environmental sensors), the mobile gateway and the nursing-care server, in advance. Next, an authentication phase is operated to secure all of the communication and data exchange among the communication entities. The proposed nursing-care support system is able to achieve the following security requirements: (a) to guarantee mutual authentication among intelligent devices, the mobile gateway and the nursing-care server; (b) to provide anonymity and un-traceability for intelligent devices; (c) to resist against forgery attack and replay attack and (d) to securely establish a robust session key between the mobile gateway and the nursing-care server.

### The registration phase of the proposed system

Before introducing our proposed system, we present the symbols and abbreviations throughout this study in Table 1. In the first stage of the registration phase, an intelligent device *d*_*i*_ (i.e. smart bio-sensors or fixed environmental sensors) sends its identity $ID_{{d_{i} }}$ to the nursing-care server *S* as a registration request. On receipt of the request from *d*_*i*_, the nursing-care server *S* generates a random number *N*_*ds*_ and uses its identity *ID*_*s*_ to compute a secret value $K_{ds} = H\left( {ID_{s} ||N_{ds} ||ID_{{d_{i} }} } \right)$. Next, the nursing-care server *S* calculates a set of un-linkable shadow identities *SID *= {*sid*_1_, *sid*_2_, …} for *d*_*i*_, where each $sid_{j} \in SID$ and $sid_{j} = H\left( {ID_{{d_{i} }} ||N_{j} ||K_{ds} } \right)$. Note that *N*_*j*_ is a random number used for deriving each *sid*_*j*_ value. Moreover, a track sequence number *Tr*_*seq*_ is generated for fast identification of intelligent device *d*_*i*_ during the authentication process as well as for preventing replay attacks. The *Tr*_*seq*_ will be stored and updated at both the nursing-care server *S* and the device *d*_*i*_ after each authentication session. In that case, the nursing-care server *S* is able to check the freshness of an incoming request from *d*_*i*_, and to achieve a fast identification of *d*_*i*_ via *Tr*_*seq*_ at the backend database during the authentication session. Finally, the nursing-care server *S* issues a security credential containing $\left( {ID_{{d_{i} }} , \, K_{ds} ,SID,Tr_{seq} ,H\left( . \right)} \right)$ to the intelligent device *d*_*i*_. At the same time, the nursing-care server will maintain a record $\left( {ID_{{d_{i} }} , \, K_{ds} ,SID,Tr_{seq} ,H\left( . \right)} \right)$ corresponding to *d*_*i*_ at the backend database. Note that *H*(.) denotes a secure one-way hash function such as SHA-3. On the other hand, the registration phase between the mobile gateway *g*_*j*_ and the nursing-care server *S* are launched in a similar way. The mobile gateway *g*_*j*_ send its identity $ID_{{g_{j} }}$ to the nursing-care server as a registration request. Next, the nursing-care server *S* calculates $K_{gs} = H\left( {ID_{s} ||N_{gs} ||ID_{{g_{j} }} } \right)$ with a newly generated random number *N*_*gs*_, and shares a security credential containing the secrets, i.e. $ID_{{g_{j} }}$ and *K*_*gs*_, with *g*_*j*_. The nursing-care server also maintains a tuple $\left( {ID_{{g_{j} }} , \, K_{gs} , \, H\left( . \right)} \right)$ corresponding to the mobile gateway *g*_*j*_ at the backend database.

**Table 1:** Notations throughout this study

Symbol	Definition
*d*_*i*_	Intelligent device (i.e. smart bio-sensors or fixed environmental sensors)
*g*_*j*_	Mobile gateway (operated by the nurse or the doctor)
*S*	The nursing-care server
$ID_{{d_{i} }}$	Private identity of *d*_*i*_
*ID*_*s*_	Public identity of the nursing-care server *S*
$ID_{{g_{j} }}$	Public identity of the mobile gateway *g*_*j*_
$AID_{{d_{i} }}$	One-time-alias identity of *d*_*i*_
*SID*	A set of un-linkable shadow identities *SID *= {*sid*_1_, *sid*_2_, …}
*K*_*ds*_	The secret key shared between *d*_*i*_ and *S*
*K*_*gs*_	The secret key shared between *g*_*j*_ and *S*
*Tr*_*seq*_	Track sequence number
*N*_*ds*_, *N*_*j*_, *N*_*gs*_, *N*_*d*_, *N*_*g*_, *m*	Random numbers
*H*(.)	Secure one-way hash function, i.e. SHA-3
$\oplus$	Bitwise exclusive-or operation
||	Concatenation operation

### The authentication phase of the proposed system

**Fig. 3:**
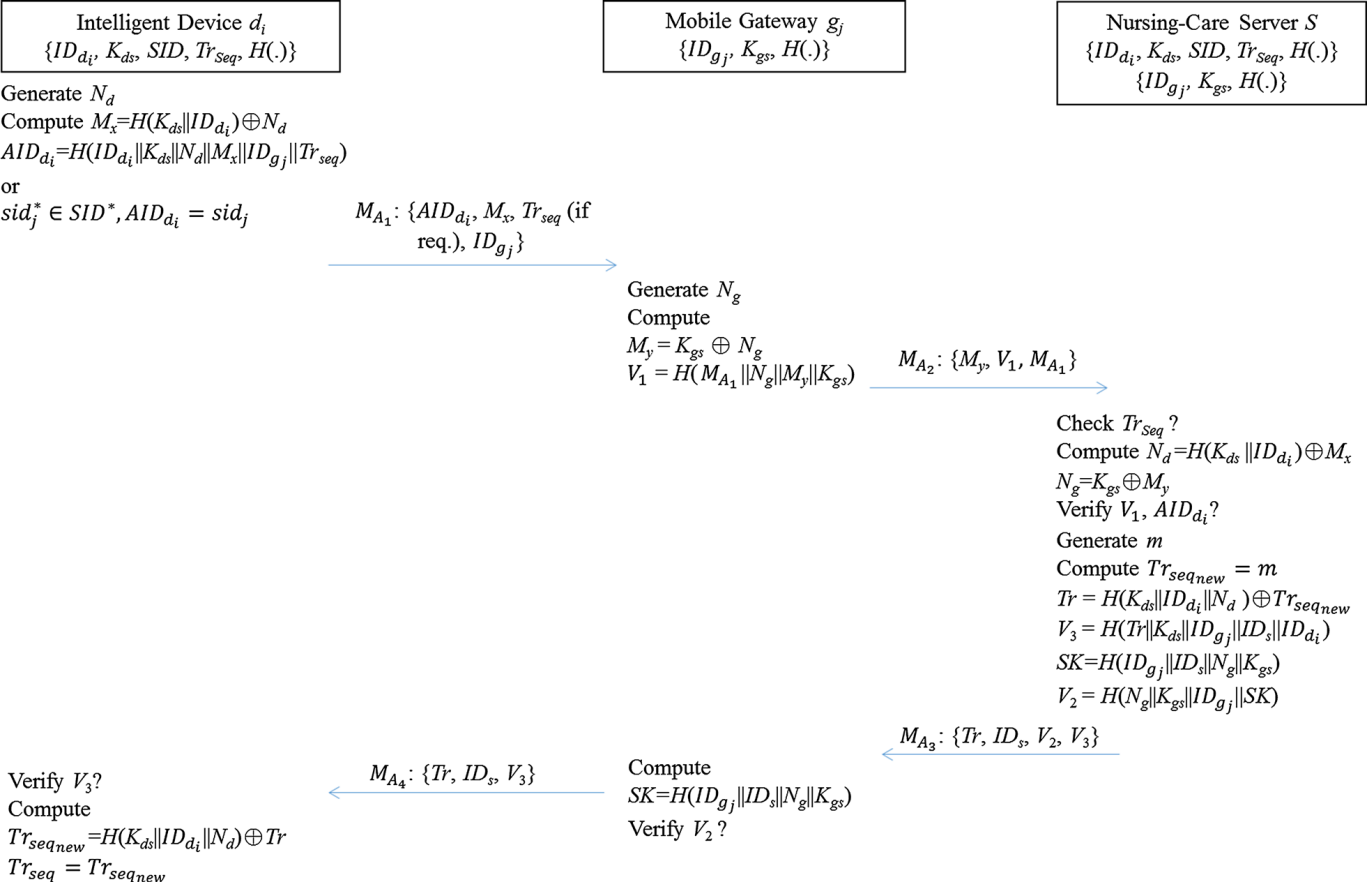
The authentication phase of the proposed IoT-based nursing-care support system

In the authentication phase, we consider that a caregiver (with a mobile gateway) would like to provide on-demand nursing care services to patients via contactless and real-time data collection and analysis mechanisms. Under the insecure IoT communication architecture, an authentication procedure is needed to establish a secure communication channel for data exchange among intelligent devices, the mobile gateway and the nursing-care server. The detailed communication procedures of the authentication phase are as shown in Fig. 3.

- Intelligent Device *d*_*i*_→ Mobile Gateway $g_{j} :M_{{A_{1} }} = \left\{ {AID_{{d_{i} }} ,\,N_{x} ,\,Tr_{seq} \left( {{\text{if req}}.} \right),\,ID_{{g_{j} }} } \right\}$
  First, the intelligent device *d*_*i*_ generates a random number *N*_*d*_ and calculates two values, i.e. $M_{x} = H\left( {K_{ds} ||ID_{{d_{i} }} } \right) \oplus N_{d}$ and $AID_{{d_{i} }} = H\left( {ID_{{d_{i} }} ||K_{ds} \left| {\left| {N_{d} } \right|} \right|M_{x} ||ID_{{g_{j} }} ||Tr_{seq} } \right)$. Next, *d*_*i*_ constructs a message $M_{{A_{1} }} = \left\{ {AID_{{d_{i} }} ,M_{x} ,Tr_{seq} ,ID_{{g_{j} }} } \right\}$ and sends $M_{{A_{1} }}$ as an authentication request to the mobile gateway *g*_*j*_. Note that, if the value *Tr*_*seq*_ shared between the intelligent device *d*_*i*_ and the nursing-care server *S* is out of synchronization, *d*_*i*_ needs to choose a fresh shadow identity *sid*_*j*_ from *SID* as the value $AID_{{d_{i} }}$. Then, *d*_*i*_ sends $M_{{A_{1} }} = \left\{ {AID_{{d_{i} }} ,M_{x} ,ID_{{g_{j} }} } \right\}$ to the mobile gateway *g*_*j*_ as an authentication request.
- Mobile Gateway *g*_*j*_→ Nursing-Care Server *S*: $M_{{A_{2} }} = \left\{ {M_{y} ,V_{ 1} ,M_{{A_{1} }} } \right\}$
  Once the mobile gateway *g*_*j*_ receives the authentication request from the intelligent device *d*_*i*_, *g*_*j*_ first generates a random number *N*_*g*_ and computes $M_{y} = K_{gs} \oplus N_{g}$ and $V_{ 1} = H\left( {M_{{A_{1} }} \left| {\left| {N_{g} } \right|} \right|M_{y} ||K_{gs} } \right)$. After that, *g*_*j*_ sends $M_{{A_{2} }} = \left\{ {M_{y} ,V_{ 1} ,M_{{A_{1} }} } \right\}$ to the nursing-care server *S*.
- Nursing-Care Server *S* → Mobile Gateway $g_{j} :M_{{A_{3} }} = \left\{ {Tr,ID_{s} ,V_{ 2} ,V_{ 3} } \right\}$
  Upon obtaining the incoming message $M_{{A_{2} }} = \left\{ {M_{y} ,V_{ 1} ,M_{{A_{1} }} } \right\}$, the nursing-care server *S* first checks whether the track sequence number *Tr*_*seq*_ is in the request or not. If it is, *S* performs step (1). If *Tr*_*seq*_ is not included in $M_{{A_{2} }}$, *S* performs step (2).
  - Step (1): Check the validity of *Tr*_*seq*_. If it holds, look for the corresponding tuple via *Tr*_*seq*_ from the backend database. Otherwise, terminate the connection. If *Tr*_*seq*_ is valid, *S* derives $N_{d} = H\left( {K_{ds} ||ID_{{d_{i} }} } \right) \oplus M_{x}$ and $N_{g} = K_{gs} \oplus M_{y}$, and then verifies *V*_1_ and $AID_{{d_{i} }}$. That is, *S* will examine (a) whether the received value *V*_1_ and the computed value $H\left( {M_{{A_{1} }} \left| {\left| {N_{g} } \right|} \right|M_{y} ||K_{gs} } \right)$ are equal or not, and (b) whether the received value $AID_{{d_{i} }}$ and the computed value $H = \left( {ID_{{d_{i} }} ||K_{ds} \left| {\left| {N_{d} } \right|} \right|M_{x} ||ID_{{g_{j} }} ||Tr_{seq} } \right)$ are equal or not. Note that if *Tr*_*seq*_ in the request does not match the one maintained in the database, the nursing-care server *S* will reject the request and terminate the connection. A new request from the device *d*_*i*_ will be asked for in which one of the fresh shadow identities *sid*_*j*_ will be picked up from the list *SID* as an anonymous identity of *d*_*i*_. In that case, the step (2) will be launched.
  - Step (2): The server *S* will verify the freshness and validity of $AID_{{d_{i} }} = sid_{j}$. If the nursing-care server *S* cannot identify the *sid*_*j*_ from the backend database, the server will terminate the connection. Next, the *S* will request the intelligent device *d*_*i*_ to try with another valid shadow identity *sid*_*j*_.

If one of the above examinations, i.e. step (1) or (2), is passed, the nursing-care server *S* will generate a random number *m*, and set $Tr_{{seq_{new} }} = m$. After that, *S* calculates $Tr = H\left( {K_{ds} ||ID_{{d_{i} }} ||N_{d} } \right) \oplus Tr_{{seq_{new} }} ,\,V_{ 3} = H\left( {Tr||K_{ds} ||ID_{{g_{j} }} ||ID_{s} ||ID_{{d_{i} }} } \right)$, $SK = H\left( {ID_{{g_{j} }} ||ID_{s} \left| {\left| {N_{g} } \right|} \right|K_{gs} } \right)$ and $V_{ 2} = H\left( {N_{g} ||K_{gs} ||ID_{{g_{j} }} ||SK} \right)$, where *SK* is a session key utilized for the next secure communication between the mobile gateway *g*_*j*_ and the nursing-care server *S*. Eventually, *S* sends $M_{{A_{3} }} = \left\{ {Tr,ID_{s} ,V_{ 2} ,V_{ 3} } \right\}$ to the mobile gateway *g*_*j*_.

- Mobile Gateway *g*_*j*_→ Intelligent Device *d*_*i*_: $M_{{A_{4} }} = \left\{ {Tr,ID_{s} ,V_{ 3} } \right\}$
  With the incoming message $M_{{A_{3} }} = \left\{ {Tr,ID_{s} ,V_{ 2} ,V_{ 3} } \right\}$, the mobile gateway *g*_*j*_ computes $SK^{\prime} = H\left( {ID_{{g_{j} }} ||ID_{s} \left| {\left| {N_{g} } \right|} \right|K_{gs} } \right)$ and $H\left( {N_{g} ||K_{gs} ||ID_{{g_{j} }} ||SK^{\prime}} \right)$, and then check if the received *V*_2_ is equal to the computed $H\left( {N_{g} ||K_{gs} ||ID_{{g_{j} }} ||SK^{\prime}} \right)$. If it holds, it is obvious that a session key *SK* is securely agreed by *g*_*j*_ and *S*. After that, the mobile gateway *g*_*j*_ sends $M_{{A_{4} }} = \left\{ {Tr,ID_{s} ,V_{ 3} } \right\}$ to the intelligent device *d*_*i*_. With $M_{{A_{4} }}$, the device *d*_*i*_ derives $H\left( {Tr||K_{ds} ||ID_{{g_{j} }} ||ID_{s} ||ID_{{d_{i} }} } \right)$ and compares it with the received *V*_3_. If these two values are identical, *d*_*i*_ computes $Tr_{{seq_{new} }} = H\left( {K_{ds} ||ID_{{d_{i} }} ||N_{d} } \right) \oplus Tr$ and sets $Tr_{seq} = Tr_{{seq_{new} }}$.

## Protocol analysis and discussions

In this section, we present a formal security analysis of the communication procedures of the proposed IoT-based nursing-care support system and discuss whether all the proposed security claims can be achieved or not.

- Claim 1: To achieve mutual authentication among communication entities in the proposed nursing-care support system

The mutual authentication via the proposed communication procedures is proven via BAN logic analysis [17, 18]. Basic constructs and logic postulates for the purpose of analysis are first presented, where the symbols *P* and *Q* are defined as principals, *X* and *Y* are defined as statements, and *K* ranges over a long-term secret.

Seven constructs is introduced as follows: (1) *P* believes *X* means that the principal *P* believes that *X* is true; (2) *P* sees *X* means that someone has sent a message containing *X* to *P*; (3) *P* said *X* denotes that *P* has actually sent a message including statement *X* at the current session of the protocol or before; (4) *P* controls *X* denotes that *P* has jurisdiction over *X*; (5) fresh(*X*) denotes that *X* has not been sent in a message; (6) $P\overset K \longleftrightarrow Q$ means that the secret *K* is shared between the principals *P* and *Q*, and (7) {*X*}_*K*_ means that the *X* is encrypted or protected under the key *K*. Next, we presented five major rules as logical postulates. First, in the message-meaning rule (referred to rule 1), we believe that if *P* believes $P\overset K \longleftrightarrow Q$ and *P* sees {*X*}_*K*_, then we postulate *P* believes *Q* said *X*. Second, the nonce-verification rule (referred to rule 2) denotes that if *P* believes fresh (*X*) and *P* believes *Q* said *X*, then we postulate *P* believes *Q* believes *X*. Third, the jurisdiction rule (referred to rule 3) means that if *P* believes *Q* controls *X* and *P* believes *Q* believes *X*, then we postulate *P* believes *X*. Fourth, a rule 4 is identified for that if *P* sees (*X*, *Y*) then *P* sees *X*. In addition, if *P* believes $P\overset K \longleftrightarrow Q$ and *P* sees {*X*}_*K*_, then *P* sees *X*. Fifth, the final rule 5 denotes that if one part of a formula is fresh, then the entire formula must also be fresh. If *P* believes fresh (*X*), then *P* believes fresh (*X*, *Y*). Finally, we demonstrate seven assumptions of our proposed system in the following.

- Assumption 1: *d*_*i*_, *S* believe $$d_{i} \overset {ID_{{d_{i} }} , K_{ds} , SID, Tr_{seq} } \longleftrightarrow S$$
- Assumption 2: *g*_*j*_, *S* believe $$g_{j} \overset {ID_{{g_{i} }} , K_{gs} } \longleftrightarrow S$$
- Assumption 3: *d*_*i*_, *S* believe fresh(*N*_*d*_)
- Assumption 4: *g*_*j*_, *S* believe fresh(*N*_*g*_)
- Assumption 5: *d*_*i*_ believes fresh(*m*)
- Assumption 6: *d*_*i*_ believes *S* controls *N*_*d*_
- Assumption 7: *g*_*j*_ believes *S* controls *N*_*g*_

Before the security analysis, we conduct the concrete realization of our proposed communication procedures as follows:

Step 1: $d_{i} \to g_{j} :\left\{ {AID_{{d_{i} }} ,M_{x} ,Tr_{seq} \left( {{\text{if req}}.} \right),ID_{{g_{j} }} } \right\}$, where $AID_{{d_{i} }} = H\left( {ID_{{d_{i} }} ||K_{ds} \left| {\left| {N_{d} } \right|} \right|M_{x} ||ID_{{g_{j} }} ||Tr_{seq} } \right)$ and $M_{x} = H\left( {K_{ds} ||ID_{{d_{i} }} } \right) \oplus N_{d} .$

Step 2: $g_{j} \to S:\left\{ {M_{y} , \, V_{ 1} ,AID_{{d_{i} }} ,M_{x} ,Tr_{seq} \left( {{\text{if req}}.} \right),ID_{{g_{j} }} } \right\}$, where $M_{y} = K_{gs} \oplus N_{g}$ and $V_{1} = H\left( {M_{{A_{1} }} \left| {\left| {N_{g} } \right|} \right|M_{y} ||K_{gs} } \right).$

Step 3: $S \to g_{j} :\left\{ {Tr,ID_{s} ,V_{ 2} ,V_{ 3} } \right\}$, where $Tr = H\left( {K_{ds} ||ID_{{d_{i} }} ||N_{d} } \right) \oplus Tr_{{seq_{new} }} ,V_{ 2} = H\left( {N_{g} ||K_{gs} ||ID_{{g_{j} }} ||SK} \right)$, $V_{ 3} = H\left( {Tr||K_{ds} ||ID_{{g_{j} }} ||ID_{s} ||ID_{{d_{i} }} } \right)$ and $SK = H\left( {ID_{{g_{j} }} ||ID_{s} \left| {\left| {N_{g} } \right|} \right|K_{gs} } \right)$.

Step 4: $g_{j} \to d_{i} :\left\{ {Tr,ID_{s} ,V_{ 3} } \right\}$, where $Tr = H\left( {K_{ds} ||ID_{{d_{i} }} ||N_{d} } \right) \oplus Tr_{{seq_{new} }}$ and $V_{ 3} = H\left( {Tr||K_{ds} ||ID_{{g_{j} }} ||ID_{s} ||ID_{{d_{i} }} } \right).$

After that, the following steps show that the formal analysis of the mutual authentication:

1. *g*_*j*_ sees{*ID*_*s*_, *V*_2_} (Step 3).
2. *g*_*j*_ believe $$g_{j} \overset {ID_{{g_{i} }} , K_{gs} } \longleftrightarrow S$$(Assumption 2).
3. *g*_*j*_ believes *S* said {*ID*_*s*_, *V*_2_} [(1) and (2), inferred by Rule 1].
4. *g*_*j*_ believes fresh(*N*_*g*_) (Assumption 4).
5. *g*_*j*_ believes *S* believes {*ID*_*s*_, *V*_2_} [(3) and (4), inferred by Rule 2].
6. *g*_*j*_ believes *S* controls{*N*_*g*_} (Assumption 7).
7. *g*_*j*_ believes {*ID*_*s*_, *V*_2_} [(5) and (6), Inferred by Rule 3].
8. *d*_*i*_ sees {*Tr*, *ID*_*s*_, *V*_3_} (Step 4).
9. *d*_*i*_ believes $$d_{i} \overset {ID_{{d_{i} }} , K_{ds} , SID, Tr_{seq} } \longleftrightarrow S$$ (Assumption 1).
10. *d*_*i*_ believes *S* said {*Tr*, *ID*_*s*_, *V*_3_} [(8) and (9), Inferred by Rule 1].
11. *d*_*i*_ believes fresh(*N*_*d*_), fresh(*m*) (Assumption 3 and 5).
12. *d*_*i*_ believes *S* believes {*Tr*, *ID*_*s*_, *V*_3_} [(10) and (11), Inferred by Rule 2].
13. *d*_*i*_ believes *S* controls {*N*_*d*_} (Assumption 6).
14. *d*_*i*_ believes {*Tr*, *ID*_*s*_, *V*_3_} [(12) and (13), inferred by Rule 3].

So far, we obtain the following results.

*g*_*j*_ believes *S* believes {*ID*_*s*_, *V*_2_} [From (5)]

*g*_*j*_ believes {*ID*_*s*_, *V*_2_} [From (7)]

*d*_*i*_ believes *S* believes {*Tr*, *ID*_*s*_, *V*_3_} [From (12)]

*d*_*i*_ believes {*Tr*, *ID*_*s*_, *V*_3_} [From (14)]

Based on the assumption of the trustworthiness of *S* and the four results (5), (7), (12) and (14), both *d*_*i*_ and *g*_*j*_ can authenticate with each other via *S*.

- Claim 2: To guarantee anonymity and un-traceability for each intelligent device in the proposed nursing-care support system

During the communication procedures of the proposed system, two random numbers *N*_*d*_ and *N*_*g*_ are utilized to randomize the messages, such as $AID_{{d_{i} }} ,M_{x} ,M_{y} ,V_{ 1} ,Tr,V_{ 2} \,{\text{and}}\,V_{ 3}$, transmitted among the intelligent devices *d*_*i*_, the mobile gateway *g*_*j*_ and the nursing-care server *S*. The *g*_*j*_ and *S* cannot obtain the real identity of *d*_*i*_. In other words, the identity $ID_{{d_{i} }}$ is included in a randomized cipher text during each communication session. Therefore, we can claim that our proposed system can provide the anonymity, and the un-traceability can be guaranteed also. On the other hand, the shadow identity scheme in our system is adopted to deal with the condition of loss of synchronization between the intelligent device and the nursing-care server. Since the shadow identity is randomly chosen, it does not provide any clue for malicious attacks due to the un-linkable property of them.

- Claim 3: To resist against forgery attack and replay attack

Adversaries may counterfeit messages to deceive the legal communication entities, i.e. *d*_*i*_, *g*_*j*_ and *S*. However, without *N*_*d*_, *N*_*g*_, *K*_*ds*_ and *K*_*gs*_, it is hard to create a counterfeited but legitimate messages such as $\left\{ {M_{y} , \, V_{ 1} ,AID_{{d_{i} }} ,M_{x} ,Tr_{seq} \left( {{\text{if req}}.} \right),ID_{{g_{j} }} } \right\}$ and {*Tr*, *ID*_*s*_, *V*_2_, *V*_3_} for spoofing. Even if the adversaries launch a replay attack with a previously eavesdropped message, the previously-used message cannot be successfully verified. This is because the random numbers *N*_*d*_ and *N*_*g*_ must be fresh and on-time valid at each session. As a result, we can claim that the resistance to forgery attack and replay attack can be guaranteed in our proposed system.

- Claim 4: To preserve data confidentiality via a secure transmission channel established between the mobile gateway and the nursing-care server

During the transmission of the proposed system, all of the messages $\left\{ {M_{y} ,V_{1} ,AID_{{d_{i} }} ,M_{x} ,Tr_{seq} \left( {{\text{if req}}.} \right),ID_{{g_{j} }} } \right\}$ and {*Tr*, *ID*_*s*_, *V*_2_, *V*_3_} are protected through a secure one-way hash function (e.g., SHA-3), and two robust secrets *K*_*ds*_ and *K*_*gs*_ chosen by *S*. Without *K*_*ds*_ and *K*_*gs*_, it is difficult to retrieve any useful information from transmitted cipher texts owing to the irreversibility of the one-way hash function. The proposed system thus provides data confidentiality. Moreover, according to the analysis of Claim 1, the mutual authentication of *g*_*j*_ and *S* is achieved via *V*_2_. It is obvious that a session key *SK* can securely be agreed by *g*_*j*_ and *S* during our proposed authentication phase, and this session key *SK* will then be exploited on the verification of *V*_2_. Therefore, we argue that a robust channel will be established between the mobile gateway and the nursing-care server for secure communication.

## Performance evaluation and results

To evaluate the practicability of the proposed IoT-based nursing-care support system, we implement a demo system as a proof-of-concept and evaluate it’s performance. The implementation was established under the platform shown in Table 2, where the Raspberry PI II platform is simulated as an intelligent device operating with a smartphone (as the mobile gateway) and a desktop computer (as the nursing-care server). Because a performance bottleneck occurring at the intelligent device is always in a high probability when compared to a smartphone and a desktop computer, in this implementation we focus on the performance evaluation of the intelligent device. A secure one-way hash function, i.e. SHA-3 (512 bits), and bitwise exclusive-or operation are adopted. In addition, in our system implementation, “$ID_{{d_{i} }}$, *ID*_*s*_, $ID_{{g_{j} }}$, *K*_*ds*_ and *K*_*gs*_” are set to 96-bits and “*SID*, *Tr*_*seq*_, *N*_*ds*_, *N*_*j*_, *N*_*gs*_, *N*_*d*_, *N*_*g*_ and *m*” are set to 64-bits. Each time *SID* contains 100 *sid*_*j*_ values. The experiments are implemented via Oracle Java 8 and Eclipse 3.8, and we implement the SHA-3 hash function with the support of Bouncy Castle Crypto APIs [19].

**Table 2:** Implementation environment

Environment	Description
Raspberry PI II	Broadcom BCM2836 @ 1 GHz Quad-Core ARM Cortex-A7 Architecture, 1 GB DDR2 RAM and SanDisk 16 GB Class 10 SD Card
Operating system	Raspbian 2016/03
Programming IDE	Eclipse 3.8 with Oracle Java 8 ARM
Crypto API	The Bouncy Castle Crypto APIs
Environment	Description

Table 3 presents the computation cost required in our proposed IoT-based nursing-care support system. During the registration phase, we need to perform (2 + *k*) times of random number generation and (2 + *k*) times of one-way hash function. Note that *k* is the size of *SID* and in our implementation we set *k* as 100. In that case, in the registration phase we have to execute 102 times the random number generation and 102 times that of the one-way hash function, and we found that the total computation time is around 14.23 ms. Note that 7.09 ms is needed for 102 RN and 7.14 ms is required for 102 H. Next, in the authentication phase we require the execution time of 6.33 ms and 5.43 ms, respectively, to perform all of the cryptographic modules, such as random number generations, exclusive-or operations and the SHA-3 (512-bits) hash function, during a normal communication session. Our implementation presents that the computation cost will be majorly dominated by the SHA-3 hash function as the execution time of the random number generation and exclusive-or operation are comparatively slight. The execution time to perform all of the SHA-3 functions required in our proposed system takes around 94% of the total computation cost. It is obvious that the SHA-3 function may become a bottleneck in terms of the performance points when the scale of the network becomes larger. Note that, in our system implementation, the input bit sequences of SHA-3 function are 192 bits, 288 bits, 800 bits, 896 bits, 992 bits and 1728 bits.

**Table 3:** Execution time of the proposed IoT-based nursing-care support system

Phase	Computation cost	Execution time (ms)
Registration	(2 + *k*) RN + (2 + *k*) H^a^	14.23 ms (i.e. 102RN + 102H)
Authentication	3RN + 6XOR + 14H (with *Tr*_*seq*_)	6.33 ms (i.e. 3RN + 6XOR + 14H)
	3RN + 6XOR + 12H (without *Tr*_*seq*_)	5.43 ms (i.e. 3RN + 6XOR + 12H)

RN means random number

XOR means bitwise exclusive-or operation

H means the one-way hash function SHA-3 (512 bits)

^a^*k* is the size of *SID* which contains *ksid*_*j*_ values

## Conclusions

In this paper, we present an efficient IoT-based nursing-care service system to support the caregiver (such as nurse/doctor/administrator) to provide better quality in nursing care activities. In consideration of the trade-off between system security and computation efficiency, we adopt lightweight cryptographic modules as the major data protection technique in the communication procedures of our proposed nursing-care support system. A demo system is implemented as a proof of concept to show the practicability of the proposed method in which a reasonable and user-acceptable computation cost, i.e. at most 6.33 ms, is presented. Moreover, based on the analysis we conducted, the security robustness of the proposed nursing-care support system is guaranteed. In brief, we argue that our proposed system is very suitable for IoT-based environments and will be a highly competitive candidate for the next generation of nursing-care service systems.

## Abbreviations

IoT: Internet-of-Things
BLE: Bluetooth Low Energy
NFC: near-field communication
RFID: radio frequency identification
SHA: Secure Hash Algorithm
6LoWPAN: IPv6 over Low-Power Wireless Personal Area Networks
ASSET: adaptive security for smart Internet of Things in e-health
BSN: body sensor networks
